# Increased inflammation burden index increasing the risk of poor prognosis in patients with chronic kidney disease in NHANES study

**DOI:** 10.1080/0886022X.2025.2523574

**Published:** 2025-07-07

**Authors:** Xinhao Chen, Dongming Ye, Weijie Zhou, Ziyi Huang, Wanwen Zou, Rongjun Zeng, Wenyong Chen

**Affiliations:** a Department of Nephrology, The Sixth Affiliated Hospital of Jinan University, Dongguan, China; b Department of Urology, The Sixth Affiliated Hospital of Jinan University, Dongguan, China

**Keywords:** Inflammation burden index, chronic kidney disease, inflammation status, mortality

## Abstract

**Objective:**

Several studies illustrated high level of inflammation is an important factor affecting the poor prognosis of patients with chronic kidney disease. Inflammatory burden index (IBI) is a composite maker by incorporating a variety of inflammatory markers to facilitate more in-depth assessment for systemic inflammation. The purpose of this research is to examine the correlation between IBI and prognosis in CKD population of American adults.

**Methods:**

This study included CKD patients recruited from the U.S. National Health and Nutrition Examination Survey (NHANES) database from 1999 to 2010. The primary endpoints were identified as all-cause and cardiovascular mortality, with a median follow-up period of 83 (IQR 83–172) months. Associations between IBI and endpoints were explored using multivariable in-depth regressions, restricted cubic splines (RCS) and subgroup analyses.

**Results:**

The research enrolled 3,975 subjects. During the follow-up period, there were 2,016 all-cause deaths and 628 cardiovascular deaths. Participants were divided into four groups based on IBI quartiles. Fully adjusted multivariate Cox regression analysis revealed increased IBI was associated with increased both all-cause and cardiovascular mortality risk [Quartile 4 vs. Quartile 1: all-cause mortality, HR = 1.37 (1.21–1.55); cardiovascular mortality, HR = 1.47 (1.17–1.84)]. The RCS analysis further supported a positive relationship between IBI and both endpoints.

**Conclusions:**

The IBI is strong correlated to the mortality of CKD individuals. Elevated levels of inflammation increased the risk of adverse clinical endpoints.

## Introduction

The increasing prevalence and mortality of chronic kidney disease (CKD) with characteristic of continuous renal function deterioration posed a serious public health challenge around the world [1]. Chronic systemic inflammation was proven to significantly contribute to progression of CKD, potential mechanisms including endothelial dysfunction, oxidative stress, as well as immune dysregulation [2]. Inflammation was considered as one of key factor in both the initiation and development of CKD. Recent evidence has increasingly connected persistent low-grade inflammation to elevated clinical adverse event risk among CKD patients [3,4], with cardiovascular disease (CVD) being one of the most important and severe causes of death [5–7]. This underscores the urgent need for effective and practical prognostic biomarkers to help reduce CKD-related mortality and inform therapeutic interventions.

Previous studies demonstrated C-reactive protein (CRP) level was a powerful maker of adverse outcome among CKD population [8]. However, the correlation between CRP and renal function decline remain inconclusive [9]. Moreover, CRP has been regarded as a relatively unspecific acute phase protein, particularly among dialysis patients who experience a variety of inflammatory challenges, such as biocompatible membranes. This can lead to substantial fluctuations in CRP levels [10]. To more comprehensively assess inflammatory-immune status, new composite indices are needed for better predictive accuracy.

The inflammatory burden index (IBI) is a composite maker with the potential of improving diagnostic accuracy by incorporating a variety of inflammatory markers to facilitate more rounded evaluation for systemic inflammation [11]. Previous research has shown that the IBI has superior predictive validity for clinical events than any single marker [11,12]. So far, the relation of IBI and prognosis among CKD patients has not been clarified. Being an emerging biomarker, IBI holds significant potential for research and clinical application in CKD. By conducting an in-depth study on the relation of IBI and clinical adverse prognosis risk, new insights and methods can be provided for the treatment and prognosis evaluation of CKD patients.

Therefore, this study aimed to examine the association between the IBI and the risks of all-cause and cardiovascular mortality among patients CKD using data from the National Health and Nutrition Examination Survey (NHANES). Understanding this relationship may help clarify the role of inflammation in mortality risk and support the development of targeted therapeutic strategies.

## Methods

### Study database and population

**Figure 1.**
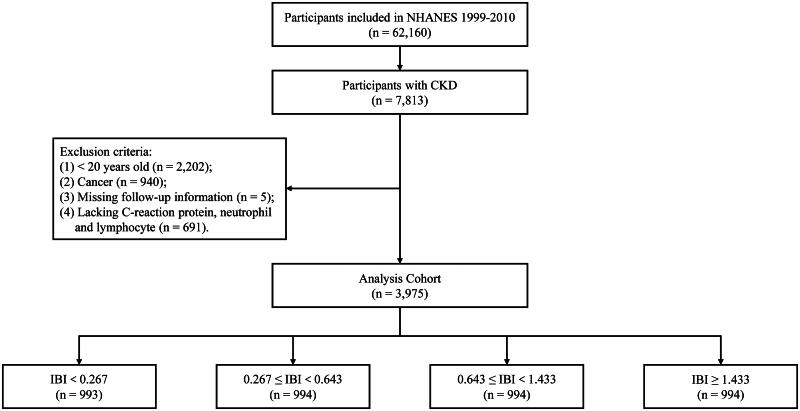
Study flow chart. Abbreviation: NHANES, National Health and Nutrition Examination Survey; CKD, chronic kidney disease; IBI, inflammatory burden index.

The NHANES is a series of nationwide cross-sectional surveys conducted by the Center of Disease Control and Prevention to assess the health and nutritional status of adults and children in the United States [13]. Stratified, multistage, clustered probability sampling design was utilized to guarantee nationally representative. This survey is conducted annually, and public-use data are released in a 2-year cycle. The complete survey was composed of a structured interview implemented in home, followed by a standardized health checkup including questionnaires, physical examination, and laboratory tests at a mobile examination center (MEC) [14]. All procedures and contents were approved by the National Center for Health Statistics (NCHS) Ethic Review Board, and written informed consents were obtained from all participants. Currently, only participants in NHANES 1990–2010 have CRP tested, thus the data from these six survey cycles were used in the current analysis. Participants with CKD were enrolled. Exclusion criteria incorporated the following [1]: patients < 20 years old (*n* = 3,605) [2]; patients diagnosed with cancer (*n* = 1,644) [3]; patients without available follow-up information (*n* = 9) [4]; patients with incomplete records of certain markers, including CRP, neutrophil and lymphocyte (*n* = 3,884). The study procedure was shown in Figure 1. The NHANES study protocol was approved by the NCHS Research Ethics Review Board. Written informed consent was obtained from all participants.

### Primary endpoint and definitions

Primary endpoints of this research were identified as all-cause and cardiovascular mortality. Mortality and follow-up information was obtained by probabilistic matching of NHANES data with the National Death Index (NDI) (https://www.cdc.gov/) through the use of personally identifiable information. Follow-up periods began on the NHANES questionnaire date and lasted until the date of death or December 31, 2018, depending on which occurred first.

CKD was defined as estimated glomerular filtration rate (eGFR) less than 60 mL/min/1.73m^2^ or urine albumin creatine ratio (uACR) more than 30 mg/g [15,16]. The level of eGFR was accessed on the basis of the Chronic Kidney Disease Epidemiology Collaboration equation (CKD-EPI equation) [17]. The IBI is formulated as a composite index that combines a variety of inflammatory indicators, including CRP, neutrophil and lymphocyte, which was calculated using the formula: CRP (mg/dL) × neutrophil (10^9^/L)/lymphocyte (10^9^/L). CRP was detected as standard CRP (variable name: LBXCRP). Elevated IBI scores were regarded to be correlated with increased inflammatory burden. Based on IBI scores, patients were categorized into four quartile groups, where the first quartile showed the lowest inflammatory burden and the fourth quartile showed the highest (Quartile 1: IBI ≤ 0.267; Quartile 2: 0.267 ≤ IBI < 0.643; Quartile 3: 0.643 ≤ IBI < 1.433; Quartile 4: IBI ≥ 1.433).

### Statistical analysis

Continuous variables were showed as mean (SD) for normally distributed data and median (IQR) for non-normal distribution data. Categorical information was presented in terms of counts and percentages. Characteristics across groups were assessed through ANOVA, Kruskal Wallis tests and chi-squared tests, where applicable. The cumulative hazard of clinical endpoints was analyzed through Kaplan-Meier analysis and the log-rank test.

The correlation between IBI value and clinical endpoints was analyzed by Cox regression models. Potential confounding variables were chosen according to significant baseline differences or clinical relevance. Four regression models were constructed to evaluate the hazard ratio. The regression models include Model 1 (unadjusted); Model 2 (adjusted for age and sex); Model 3 [further adjusted for body mass index (BMI), race and education]; Model 4 [further adjusted for congestive heart failure (CHF), coronary artery disease (CAD), hypertension, diabetes mellitus (DM) and stroke]; Model 5 [further adjusted for CKD uACR classification based on Model 4]; Model 6 [further adjusted for CKD eGFR classification based on Model 4]. We conducted restricted cubic spline (RCS) analysis with the same covariates to test the potential nonlinear association. To explore the association between the IBI and primary endpoints (all-cause and cardiovascular mortality) across different groups, subgroup analyses were performed based on sex (male and female), CHF (yes and no), CAD (yes and no), hypertension (yes and no), DM (yes and no), and stroke (yes and no). Sensitivity analyses were performed in participants excluding those with dialysis. All statistical analyses were conducted using R version 4.2.2 (R Foundation for Statistical Computing, Vienna, Austria). Statistically significant was defined as a *P* value of <0.05.

## Results

### Baseline characteristics

In total, 3,975 patients with CKD (mean age, 63.58 ± 17.34 years; 53.38% females) were enrolled in the study. Non-Hispanic white (49.66%) comprised the majority of the study population. The overall mean IBI score for the cohort was 1.11 and the mean BMI was 29.75 ± 7.01 kg/m^2^. Of the patients, 3,159 patients (79.47%) had hypertension, 1,454 patients (36.99%) had DM, 613 patients (15.57%) had CAD, and 342 patients (8.69%) had CHF.

In addition, compared to the lowest IBI quartile, higher IBI quartile showed larger proportions of patients with comorbidities such as hypertension (83.00 vs. 80.89% vs. 78.87 vs. 75.13%, *p* < 0.001), DM (43.09 vs. 37.86% vs. 36.46 vs. 30.67%, *p* < 0.001) and CHF (11.81 vs. 9.84% vs. 7.62 vs. 5.49%, *p* < 0.001). Baseline data were illustrated in Table 1.

**Table 1.** Baseline characteristics.

Characteristics	Overall	Quartile 1	Quartile 2	Quartile 3	Quartile 4	*P* value
	(*N* = 3,975)	(*N* = 993)	(*N* = 994)	(*N* = 994)	(*N* = 994)	
**Demographic**						
Age, years	63.58 (17.34)	63.43 (18.07)	63.83 (17.12)	64.48 (17.16)	62.57 (16.95)	0.10
Female, sex, *n* (%)	2,122 (53.38)	518 (52.17)	511 (51.41)	528 (53.12)	565 (56.84)	0.07
BMI, kg/m^2^	29.75 (7.01)	27.10 (5.49)	29.21 (6.17)	30.41 (6.44)	32.36 (8.56)	<0.001
Race, *n* (%)						0.15
Non-hispanic white	1,974 (49.66)	497 (50.05)	486 (48.89)	497 (50.00)	494 (49.70)	
Non-hispanic black	818 (20.58)	216 (21.75)	197 (19.82)	197 (19.82)	208 (20.93)	
Mexican American	782 (19.67)	165 (16.62)	203 (20.42)	210 (21.13)	204 (20.52)	
Other	401 (10.09)	115 (11.58)	108 (10.87)	90 (9.05)	88 (8.85)	
Education, *n* (%)						0.06
Below high school	1,621 (40.86)	368 (37.13)	410 (41.29)	420 (42.30)	423 (42.73)	
High school	950 (23.95)	238 (24.02)	255 (25.68)	228 (22.96)	229 (23.13)	
Over high school	1,396 (35.19)	385 (38.85)	328 (33.03)	345 (34.74)	338 (34.14)	
**Comorbidity**						
CHF, *n* (%)	342 (8.69)	54 (5.49)	75 (7.62)	97 (9.84)	116 (11.81)	<0.001
CAD, *n* (%)	613 (15.57)	145 (14.69)	144 (14.66)	158 (16.02)	166 (16.90)	0.44
Hypertension, *n* (%)	3,159 (79.47)	746 (75.13)	784 (78.87)	804 (80.89)	825 (83.00)	<0.001
Diabetes mellitus, *n* (%)	1,454 (36.99)	303 (30.67)	362 (36.46)	371 (37.86)	418 (43.09)	<0.001
Stroke, *n* (%)	370 (9.34)	91 (9.17)	99 (10.01)	81 (8.17)	99 (10.00)	0.45
CKD grade						
uACR classification						0.003
A1	1,168 (30.20)	317 (32.45)	307 (31.39)	286 (29.79)	258 (27.07)	
A2	2,236 (57.81)	568 (58.14)	549 (56.13)	568 (59.17)	551 (57.82)	
A3	464 (12.00)	92 (9.42)	122 (12.47)	106 (11.04)	144 (15.11)	
eGFR classification						0.01
G1	1,142 (28.87)	294 (29.82)	277 (28.04)	267 (27.00)	304 (30.65)	
G2	919 (23.24)	218 (22.11)	241 (24.39)	250 (25.28)	210 (21.17)	
G3a	1,258 (31.81)	338 (34.28)	316 (31.98)	296 (29.93)	308 (31.05)	
G3b	444 (11.23)	102 (10.34)	114 (11.54)	122 (12.34)	106 (10.69)	
G4	136 (3.44)	27 (2.74)	32 (3.24)	37 (3.74)	40 (4.03)	
G5	56 (1.42)	7 (0.71)	8 (0.81)	17 (1.72)	24 (2.42)	
Dialysis	49 (1.23)	10 (1.01)	12 (1.21)	13 (1.31)	14 (1.41)	0.87
**Laboratory test**						
IBI	1.11 (1.24)	0.16 (0.06)	0.43 (0.11)	0.97 (0.22)	2.88 (1.25)	<0.001
CRP, mg/dL	0.47 (0.51)	0.10 (0.07)	0.23 (0.12)	0.45 (0.22)	1.09 (0.62)	<0.001
Neutrophil,10^9^/L	4.48 (1.85)	3.72 (1.29)	4.22 (1.38)	4.68 (1.63)	5.30 (2.49)	<0.001
Lymphocyte, 10^9^/L	2.06 (1.12)	2.18 (1.73)	2.11 (0.94)	2.04 (0.77)	1.93 (0.73)	<0.001
Creatinine, μmol/L	102.49 (83.11)	99.21 (80.07)	99.66 (66.15)	104.86 (90.11)	106.20 (93.25)	0.14
eGFR, mL/min/1.73 m²	71.41 (29.34)	72.65 (29.00)	71.30 (28.42)	69.98 (28.85)	71.72 (31.00)	0.24
uACR, mg/g	235.44 (882.50)	179.54 (756.42)	247.62 (922.69)	238.02 (957.33)	277.64 (880.23)	0.10
Total cholesterol, mmol/L	5.20 (1.16)	5.08 (1.10)	5.22 (1.23)	5.26 (1.11)	5.23 (1.18)	0.002
HDL-C, mmol/L	1.33 (0.41)	1.42 (0.42)	1.32 (0.39)	1.30 (0.40)	1.29 (0.43)	<0.001
LDL-C, mmol/L	2.97 (0.96)	2.93 (0.94)	2.99 (1.02)	3.01 (0.95)	2.95 (0.92)	0.55
Triglyceride, mmol/L	1.86 (1.37)	1.63 (1.22)	1.94 (1.44)	1.95 (1.46)	1.94 (1.31)	<0.001
HbA1c, %	6.22 (1.57)	5.96 (1.23)	6.25 (1.68)	6.25 (1.60)	6.43 (1.68)	<0.001
Albumin, g/L	4.15 (0.36)	4.26 (0.33)	4.20 (0.35)	4.14 (0.35)	4.03 (0.36)	<0.001
**Clinical outcomes**						
All-cause death	2,016 (50.72)	446 (44.91)	493 (49.60)	529 (53.22)	548 (55.13)	<0.001
Cardiovascular death	628 (15.80)	133 (13.39)	156 (15.69)	163 (16.40)	176 (17.71)	0.06

Abbreviation: BMI, body mass index; CHF, congestive heart failure; CAD, coronary artery disease; IBI, inflammatory burden index; CRP, C-reactive protein; eGFR, estimated glomerular filtration rate; uACR, urine albumin creatine ratio; HDL-C, high-density lipoprotein cholesterol; LDL-C, low-density lipoprotein cholesterol; HbA1c, hemoglobin A1c.

### Associations between IBI with mortality

**Figure 2.**
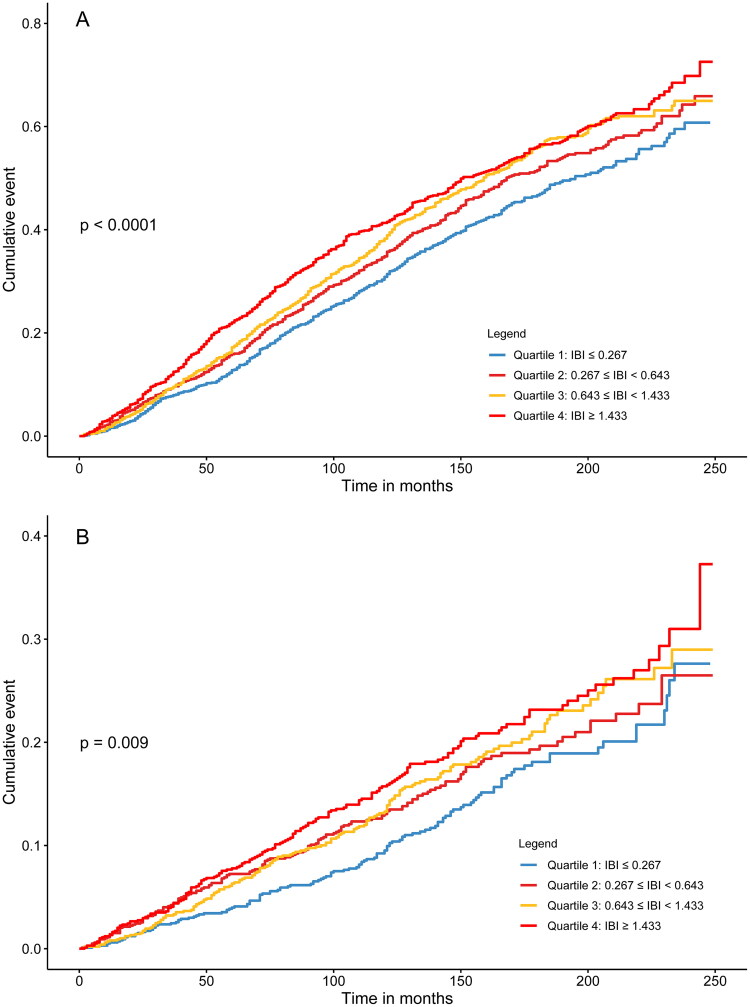
Kaplan-Meier Survival curves for all-cause mortality (A) and cardiovascular mortality (B) according to IBI. Abbreviation: IBI, inflammatory burden index.

Median follow-up duration was 131.00 months (IQR ranging from 83.00 to 172.00 months). A total of 2,016 patients (50.72%) died [Quantile 4: 548 (55.13%); Quantile 3: 529 (53.22%); Quantile 2: 493 (49.60%); Quantile 1: 446 (44.91%), *p* < 0.001]. Of these, 628 patients died due to cardiovascular disease (Quantile 4: 176 [17.71%]; Quantile 3: 163 [16.40%]; Quantile 2: 156 [15.69%]; Quantile 1: 133 [13.39%], *p* = 0.06). Moreover, Kaplan-Meier analysis demonstrated patients assigned to the highest IBI quartile showed elevated cumulative risks of all-cause and cardiovascular mortality in comparison to individuals assigned to the lowest IBI quartile (*p* < 0.001, Figure 2). Difference of renal mortality between the 4 groups were showed in Supplementary Table 1.

To investigate the relation of IBI and clinical events, different Cox proportional hazards regression models were employed. According to univariate Cox proportional hazards regression (Model 1), the risk of all-cause mortality elevated by 37% among patients who were assigned to the highest IBI quartile group (HR = 1.37; 95% CI: 1.21–1.50, *p* < 0.001), and by 18% for cardiovascular mortality (HR = 1.47; 95% CI: 1.17–1.80, *p* < 0.001). The effectiveness can still be identified upon accounting for relevant confounding factors presented in Models 2, 3, 4, 5 and 6 (Table 2). After adjusting for the CHF, CAD, DM, hypertension and stroke, patients in the higher IBI quartile group maintained a significant correlation with higher all-cause mortality risk (Quartile 4 vs. Quartile 1: HR = 1.60; 95% CI: 1.40–1.80, *p* < 0.001; Quartile 3 vs. Quartile 1: HR = 1.30; 95% CI: 1.14–1.40, *p* < 0.001; Quartile 2 vs. Quartile 1: HR = 1.19; 95% CI: 1.04–1.30, *p* = 0.01) and cardiovascular mortality (Quartile 4 vs. Quartile 1: HR = 1.67; 95% CI: 1.31–2.10, *p* < 0.001; Quartile 3 vs. Quartile 1: HR = 1.29; 95% CI: 1.01–1.60, *p* = 0.04). Tests of trends had statistical significance in each model (*P* for trend < 0.001). The results about association between IBI and risk of renal mortality were shown in Supplementary Table 2.

**Table 2.** Cox proportion hazard model stratified by 4 groups according to the IBI scores into quartiles for all-cause and cardiovascular mortality.

Model	Model 1		Model 2		Model 3		Model 4		Model 5		Model 6	
	HR (95% CI)	*P*-Value	HR (95% CI)	*P*-Value	HR (95% CI)	*P*-Value	HR (95% CI)	*P*-Value	HR (95% CI)	*P*-Value	HR (95% CI)	*P*-Value
**All-cause mortality**												
**IBI was analyzed as a continuous variable**												
Per 1-unit increment	1.09 (1.05–1.12)	<0.001	1.15 (1.12–1.19)	<0.001	1.15 (1.11–1.19)	<0.001	1.13 (1.09–1.17)	<0.001	1.12 (1.08–1.16)	<0.001	1.12 (1.08–1.16)	<0.001
**IBI was analyzed as a categorical variable**												
Quartile 1	Ref	–	Ref	–	Ref	–	Ref	–	Ref	–	Ref	–
Quartile 2	1.15 (1.02–1.31)	0.03	1.19 (1.04–1.36)	0.01	1.19 (1.04–1.36)	0.01	1.19 (1.04–1.36)	0.01	1.21 (1.06–1.39)	0.006	1.18 (1.03–1.35)	0.02
Quartile 3	1.26 (1.11–1.43)	<0.001	1.28 (1.12–1.46)	<0.001	1.28 (1.12–1.46)	<0.001	1.30 (1.14–1.49)	<0.001	1.28 (1.12–1.47)	<0.001	1.27 (1.11–1.46)	<0.001
Quartile 4	1.37 (1.21–1.55)	<0.001	1.64 (1.44–1.88)	<0.001	1.64 (1.44–1.88)	<0.001	1.60 (1.40–1.83)	<0.001	1.58 (1.37–1.81)	<0.001	1.54 (1.34–1.76)	<0.001
P for trend	<0.001		<0.001		<0.001		<0.001		<0.001		<0.001	
**Cardiovascular mortality**												
**IBI was analyzed as a continuous variable**												
Per 1-unit increment	1.09 (1.02–1.15)	0.006	1.16 (1.09–1.23)	<0.001	1.15 (1.08–1.22)	<0.001	1.13 (1.06–1.2)	<0.001	1.11 (1.04–1.18)	0.002	1.12 (1.05–1.19)	0.001
**IBI was analyzed as a categorical variable**												
Quartile 1	Ref	–	Ref	–	Ref	–	Ref	–	Ref	–	Ref	–
Quartile 2	1.22 (0.97–1.54)	0.09	1.26 (1.01–1.59)	0.015	1.27 (0.99–1.62)	0.05	1.26 (0.99–1.61)	0.042	1.24 (0.97–1.58)	0.09	1.24 (0.97–1.58)	0.09
Quartile 3	1.30 (1.03–1.63)	0.03	1.31 (1.04–1.64)	<0.001	1.28 (1.01–1.62)	0.04	1.29 (1.01–1.64)	<0.001	1.25 (0.98–1.59)	0.08	1.26 (0.98–1.6)	0.07
Quartile 4	1.47 (1.17–1.84)	<0.001	1.81 (1.44–2.27)	<0.001	1.74 (1.37–2.22)	<0.001	1.67 (1.31–2.13)	<0.001	1.58 (1.24–2.03)	<0.001	1.61 (1.26–2.06)	<0.001
P for trend	<0.001		<0.001		<0.001		<0.001		0.001		<0.001	

Abbreviation: IBI, inflammatory burden index.

Model 1: unadjusted.

Model 2: adjusted for age and sex.

Model 3: further adjusted for BMI, race and education.

Model 4: further adjusted for congestive heart failure, coronary artery disease, hypertension, diabetes mellitus and stroke.

Model 5: further adjusted for CKD uACR classification based on Model 4.

Model 6: further adjusted for CKD eGFR classification based on Model 4.

The RCS curves results indicated a non-linear association between IBI and all-cause mortality (Figure 3A–D). Combined with the changes in RCS shape, the overall risk of all-cause mortality increased with increasing value of IBI. As for cardiovascular mortality, a linear association was observed between IBI and cardiovascular mortality when adjusting for age and sex (Figure 4B). Generally, the association between IBI and risk of cardiovascular mortality showed a linear correlation (Figure 4A, C and D).

Figure 3.Restricted cubic spline regression analysis of the association between IBI and all-cause mortality.A: unadjusted; B: adjusted for age and sex; C: further adjusted for BMI, race and education; D: further adjusted for congestive heart failure, coronary artery disease, hypertension, diabetes mellitus and stroke.Abbreviation: IBI, inflammatory burden index.

**Figure:**
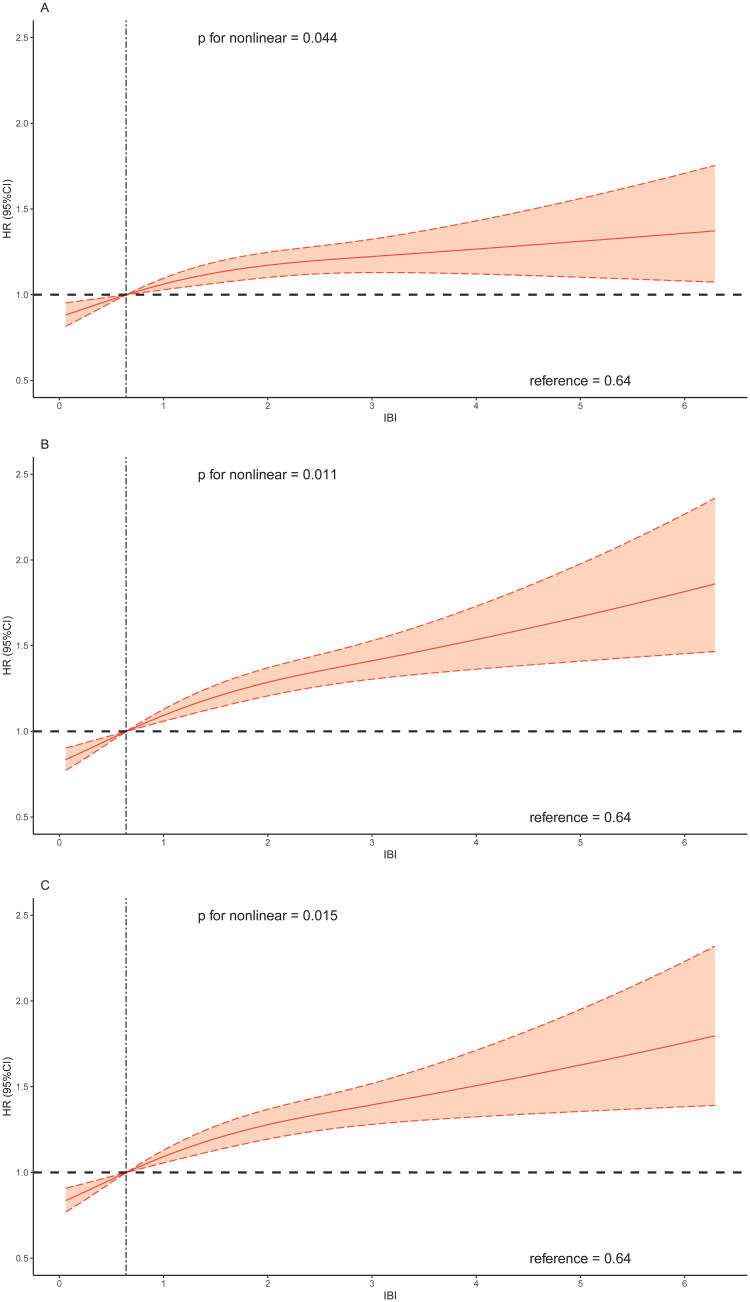

**Figure:**
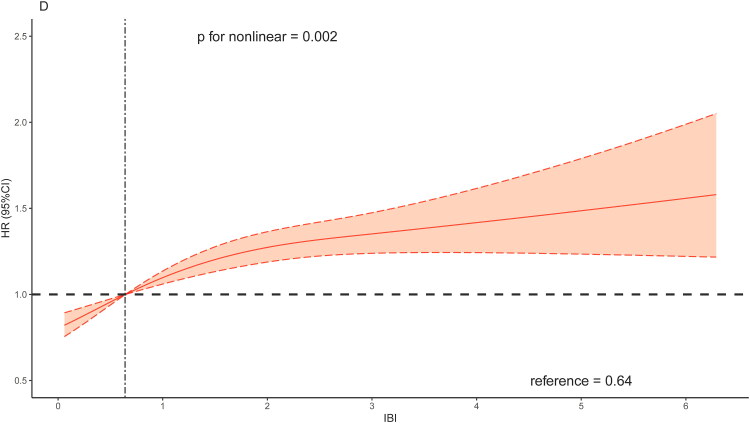

Figure 4.Restricted cubic spline regression analysis of the association between IBI and cardiovascular mortality.A: unadjusted; B: adjusted for age and sex; C: further adjusted for BMI, race and education; D: further adjusted for congestive heart failure, coronary artery disease, hypertension, diabetes mellitus and stroke.Abbreviation: IBI, inflammatory burden index.

**Figure:**
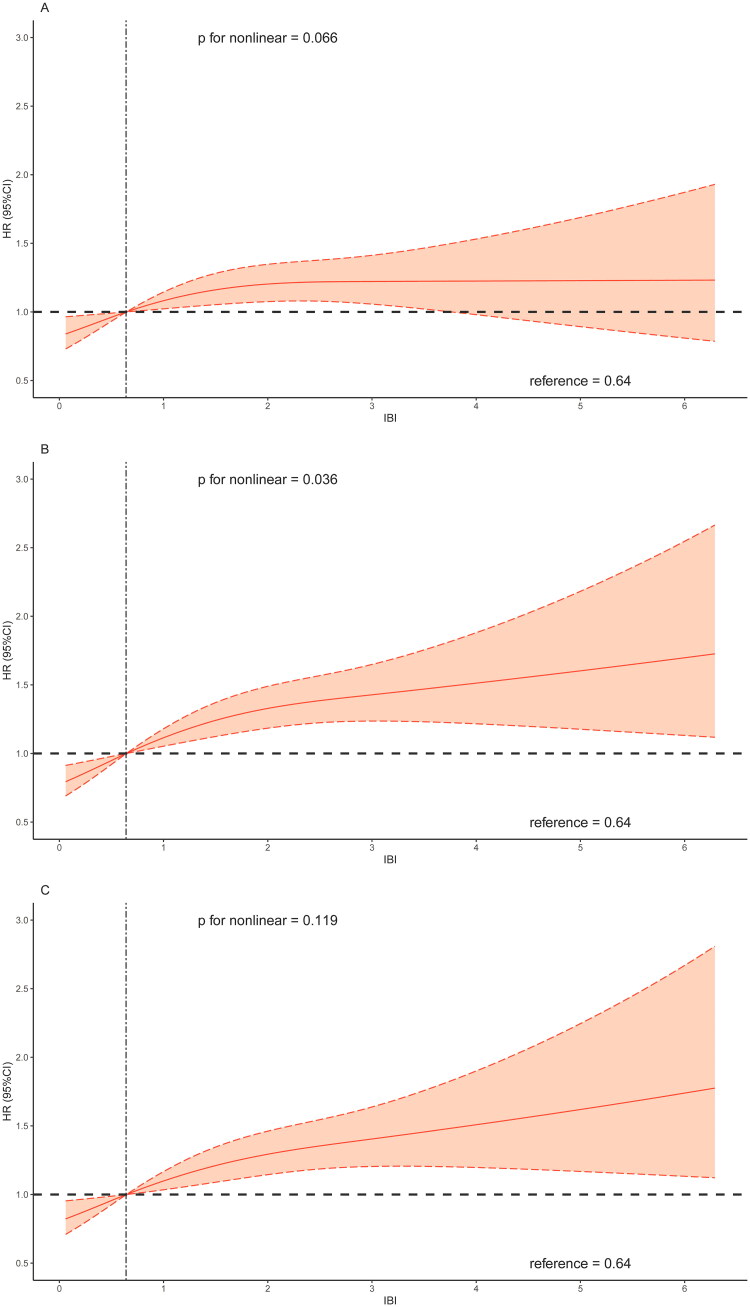

**Figure:**
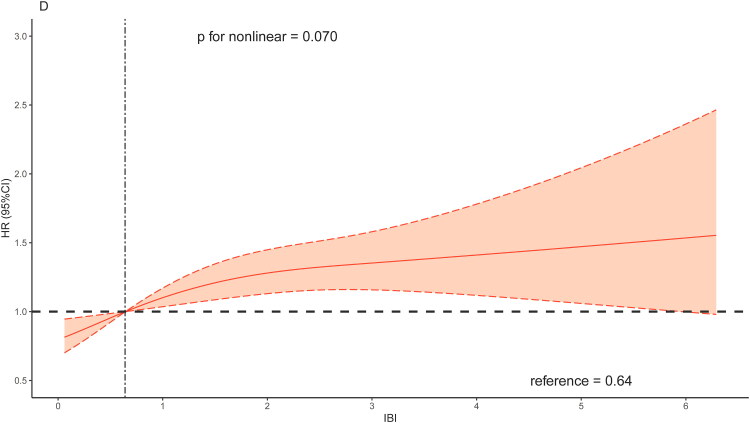

### Subgroup analyses

Stratified subgroup analyses based on factors such as sex, CHF, CAD, hypertension, DM, and stroke showed that higher IBI levels were associated with a significantly increased risk of all-cause and cardiovascular mortality in most subgroups, with no significant interaction effects observed (*P* values for interaction > 0.05) (Table 3).

**Table 3.** The association between inflammatory burden index and all-cause and cardiovascular mortality in different subgroups.

Variables	Quartile 1	Quartile 2	Quartile 3	Quartile 4	P for interaction
	HR (95% CI)	HR (95% CI)	HR (95% CI)	HR (95% CI)	
**All-cause mortality**					
Sex					0.16
Male	Reference	1.31 (1.09-1.59)	1.38 (1.14-1.66)	1.78 (1.47-2.15)	
Female	Reference	1.05 (0.87-1.28)	1.22 (1.01-1.47)	1.41 (1.16-1.71)	
Congestive heart failure					0.70
No	Reference	1.17 (1.01-1.34)	1.26 (1.09-1.45)	1.62 (1.40-1.87)	
Yes	Reference	1.44 (0.95-2.18)	1.73 (1.17-2.56)	1.64 (1.10-2.45)	
Coronary heart disease					0.36
No	Reference	1.16 (1.01-1.35)	1.26 (1.08-1.47)	1.57 (1.34-1.83)	
Yes	Reference	1.34 (1.01-1.78)	1.48 (1.12-1.96)	1.82 (1.37-2.42)	
Hypertension					0.37
No	Reference	1.02 (0.65-1.59)	1.36 (0.87-2.13)	1.93 (1.18-3.16)	
Yes	Reference	1.20 (1.04-1.39)	1.30 (1.13-1.49)	1.56 (1.36-1.80)	
Diabetes mellitus					0.13
No	Reference	1.19 (1.01-1.41)	1.21 (1.03-1.43)	1.56 (1.31-1.86)	
Yes	Reference	1.22 (0.98-1.53)	1.45 (1.16-1.81)	1.65 (1.33-2.06)	
Stroke					0.25
No	Reference	1.20 (1.04-1.40)	1.34 (1.16-1.55)	1.63 (1.40-1.89)	
Yes	Reference	1.02 (0.73-1.43)	1.11 (0.78-1.59)	1.20 (0.83-1.74)	
**Cardiovascular mortality**					
Sex					0.10
Male	Reference	1.62 (1.15-2.29)	1.52 (1.07-2.15)	2.14 (1.51-3.03)	
Female	Reference	0.96 (0.67-1.36)	1.11 (0.79-1.56)	1.31 (0.92-1.85)	
Congestive heart failure					0.28
No	Reference	1.29 (1.01-1.67)	1.18 (0.91-1.55)	1.62 (1.24-2.12)	
Yes	Reference	0.96 (0.47-1.95)	1.90 (1.04-3.47)	2.04 (1.11-3.76)	
Coronary heart disease					0.88
No	Reference	1.28 (0.96-1.70)	1.22 (0.91-1.63)	1.65 (1.23-2.20)	
Yes	Reference	1.25 (0.78-2.00)	1.54 (0.98-2.41)	1.86 (1.18-2.95)	
Hypertension					0.49
No	Reference	0.80 (0.32-1.97)	1.35 (0.58-3.11)	1.63 (0.64-4.14)	
Yes	Reference	1.30 (1.01-1.67)	1.28 (0.99-1.64)	1.64 (1.27-2.11)	
Diabetes mellitus					0.88
No	Reference	1.58 (1.16-2.14)	1.30 (0.95-1.77)	1.75 (1.27-2.41)	
Yes	Reference	0.83 (0.55-1.26)	1.27 (0.86-1.86)	1.48 (1.01-2.15)	
Stroke					0.25
No	Reference	1.18 (0.90-1.54)	1.31 (1.01-1.70)	1.65 (1.27-2.15)	
Yes	Reference	1.62 (0.91-2.90)	1.15 (0.59-2.24)	1.39 (0.71-2.74)	

### Sensitivity analyses

Sensitivity analyses showed that most results of the associations between IBI and all-cause/cause-specific mortality were robust in CKD patients without dialysis (Supplementary Table 3, 4, and 5).

### Predictive power of models with IBI and its component CRP

Supplementary Table 6 showed the predictive power of various models with IBI and its component CRP. In predicting the risk of all-cause death, the concordance index (C-index) of IBI in both categorical and continuous forms were significantly higher than that of CRP [0.773 (0.763–0.784) vs. 0.681 (0.668–0.693), *p* < 0.001; 0.773 (0.762–0.783) vs. 0.681 (0.668–0.693), *p* < 0.001]. As for the risk of cardiovascular death, the C-index of IBI in both categorical and continuous forms were also significantly higher than that of CRP [0.797 (0.779–0.814) vs. 0.712 (0.690–0.733), *p* < 0.001; 0.795 (0.778–0.813) vs. 0.712 (0.690–0.733), *p* < 0.001].

## Discussion

Our study demonstrates that higher IBI was related to increased risk of clinical endpoints including all-cause and cardiovascular death among CKD patients. We found trends of positive connection between IBI and risk of outcome events. Moreover, in subgroup analyses carried out in particular groups, such as sex, CHF, CAD, hypertension, diabetes, and stroke, the association between IBI and mortality remained robust. These results reinforce the predictive value of IBI in predicting mortality outcomes in CKD patients.

CKD was identified as a major global causes of adverse outcome in 2023 [18], affecting 15-20% population worldwide and increasing different adverse events risk [19]. Among the numerous mechanisms of CKD, systemic inflammation was a primary driver and one of the most characteristic features for uremic phenotype in advanced CKD. Several researches have reported that CRP is a powerful marker for CKD, associated with key underlying mechanisms and outcomes [20]. Series of researches demonstrated correlation between CRP levels with renal function impairment [21–24]. However, longitudinal studies have shown conflicting results. Some studies report that elevated CRP levels, representing low-grade inflammation, are related to CKD in patients who have moderate renal function impairment [8,25,26]. Similarly, a small study on IgA nephropathy found that higher CRP levels predicted kidney function decline [21]. In contrast, a randomized controlled trial indicated that increased inflammation might marginally slow down kidney function loss in CKD [27]. These inconsistent findings limit the clinical applicability of inflammatory markers and highlight the need for new inflammatory parameters to predict and intervene in CKD.

Additionally, the neutrophil-to-lymphocyte ratio (NLR) has recently been reported to be a potential predictor of micro-inflammation. Unlike single markers, NLR reduces the impact of physiological factors or sample handling on absolute blood cell counts. Researchers have found that CKD patients who have high NLR level will suffer a 1.7-fold elevating risk of adverse renal outcomes compared to those with low NLR in a Japanese cohort [28]. Other studies have also found that increased neutrophil counts and reduced lymphocyte counts predict poor outcomes in both hemodialysis and peritoneal dialysis patients [29,30]. However, these studies vary in quality, with some limited by small sample sizes and differing CKD etiologies, highlighting the need for new inflammatory markers to predict and intervene in CKD more effectively.

Given this context, IBI has emerged as a new biomarker to assess the level of inflammation with valuable practical utility. Xie et al. identified IBI as a more potent prognostic indicator for cancer compared to other inflammatory marker compositions, possibly due to its integration of CRP, neutrophils, and lymphocytes, allowing for better measurement of both acute and immune inflammation. Same findings have been revealed in prospective cohort studies on gastric and prostate cancers [11,31]. He et al. had found that higher IBI levels were associated with elevated risk of death in adults aged 45 and above using the NHANES database. Building on this, we conducted a follow-up study on adults in the U.S. NHANES database. Our findings confirm a positive link between IBI with risk of adverse clinical endpoints among CKD population. Consistent results were also obtained from subgroup analysis based on age and sex. Similarly, RCS analyses showed a positive trend between IBI and risk of outcome events. Therefore, IBI might be a novel and effective biological marker for CKD patients and have predictive value for their prognosis.

Mechanistically, our findings can be explained by the potential inflammation as well as immune dysfunction among CKD individuals. Firstly, immune activation leads to inflammation, which is a major reason of CVD. At the same time, immune suppression leads to an elevated incidence of infection, which also promotes the development of inflammation. Infections as one of the main complications account one fifth of death among CKD population [32]. Additionally, another potential reason for systemic inflammation is associated with kidney dysfunction, as it inevitably leads to fluid and solute retention, many of which may serve as pro-inflammatory uremic toxicants and cytokines [33,34]. The generation of pro-inflammatory cytokines further raises CRP levels, which is linked to increased cardiovascular risk among CKD patients [35]. The dynamic interplay between the heart and kidneys is well-established clinically. Inflammation is considered a key underlying factor in explaining the intrinsic connection between CKD and CVD. Recent studies have demonstrated that persistent inflammation is a leading contributor to both systemic and vascular aging [36]. This process may involve a decrease in intracellular glutathione levels [37], downregulation of p66Shc [38], or impairment of the antioxidant defense response mediated by NF-E2-related factor 2 (Nrf2) [39,40], all of which can exacerbate mitochondrial ROS accumulation. And in patients with chronic kidney disease, ROS production by respiratory burst enzymes is markedly elevated [41]. Chronic inflammation and oxidative stress lead to various complications such as malnutrition, phosphate-calcium imbalances, and atherosclerosis, all of which contribute to accelerated CVD progression. Research in myocardial infarction showed that the total number of neutrophils increased during the 7 days after heart attack [42]. This response is associated with the initiation of immunoreactive chemokine release and is further amplified by inflammatory mediators, including macrophage inflammatory protein-2α (MIP-2α, CXCL2, GRO-β), leukotriene B4 (LTB4), cytokine-induced neutrophil chemoattractant 1 (CINC-1, IL-8, CXCL8), and complement component 5a (C5a), which collectively enhance monocyte/macrophage signaling [43]. A cross-sectional study also reported that patients with advanced kidney failure had less peripheral blood lymphocytes compared to healthy individuals. In particular, the number of monocytes/macrophages, the main effector cells of inflammation, is increased [44]. Massive infiltration and activation of these inflammatory cells within cardiovascular system leads to an intense inflammatory response, cytokine release and further cardiomyocyte death. At the same time, bulk of classical inflammatory pathways have been proven to contribute to cardiac dysfunction and myocardial fibrosis, such as cGAS-STING, SIRT3-FOS and cAMP-AMPK pathways. Therefore, the neutrophil count increase along with the decrease in lymphocyte count might be a reasonable interpretation.

Our study provides new evidence that IBI is an independent indicator of risk of adverse clinical endpoints among CKD individuals, further reinforcing inflammation’s critical role in prognosis. By revealing the correlation between IBI and mortality risk, we offer clinical insights for more comprehensive inflammation assessments. Given the pervasive inflammatory state in CKD patients, IBI, as a composite measure of multiple inflammatory pathways, allows for more accurate prediction of mortality risk. This could provide clinicians with a valuable tool for identifying high-risk individuals as well as establishing customized interventions. This finding not only enriches our understanding of CKD management but also opens up possibilities for future research into the broader prognostic utility of IBI in CKD population.

Some limitations should also be considered. First, although our research provides important evidence on the link between IBI and death among CKD patients, the observational design of NHANES make causal inference difficult. Second, although the NHANES database is broadly representative, the long-time span of data collection and the limited follow-up period for each participant may lead to time-dependent factors affecting the results. Third, while IBI demonstrated potential as a mortality predictor in this study, its applicability in real-world clinical settings requires additional validation. Whether IBI can consistently serve as a prognostic marker for various CKD patients, especially across different racial groups, remains uncertain. This may limit the generalizability of our results, and further investigation is needed to verify our results. Fourth, NHANES survey tested CRP only 1999 to 2010. Meanwhile, the missing rate of CRP was 12.39% from 1999 to 2010. Strict inclusion and exclusion criteria were established before this study, the missing rate of CRP in our study was 13.91%. Selection bias may possible exist here. Future prospective studies with larger sample sizes are needed to verify the conclusions of this study. Fifth, because of the design of the NHANES survey, data were collected only once for each participant. It was not feasible to track the same individual in multiple cycles, so longitudinal analyses could not be performed in this study. Finally, this study only enrolled the IBI values collected from the patients at baseline interview. As the follow-up period extends, especially beyond 200 months, unknown potential confounding factors may affect the robustness of the association between IBI and risk of cardiovascular mortality. In summary, our study mainly focuses on the clinical importance of baseline IBI level on prognosis among CKD patients.

## Conclusion

Our research highlights that the IBI is a powerful and reliable maker for both all-cause and cardiovascular mortality among CKD population. The association observed between IBI and mortality risk underlines the possibilities of IBI as a novel indicator in assessing adverse events. These findings underline the essential role of inflammation in CKD outcomes, offering clinicians an additional tool for identifying high-risk patients and guiding personalized treatment strategies.

## Supplementary Material

Supplementary materials.docx

## Data Availability

The datasets generated and analyzed during the current study are available in the NHANES repository (https://wwwn.cdc.gov/nchs/nhanes/default.aspx).
