# Supplementary material for: Increased inflammation burden index increasing the risk of poor prognosis in patients with chronic kidney disease in NHANES study
Source: Ren Fail. 2025 Jul 7;47(1):2523574. doi: 10.1080/0886022X.2025.2523574 (PMC12239105; doi:10.1080/0886022X.2025.2523574)
Supplement: Supplementary materials.docx [file IRNF_A_2523574_SM8735.docx]

**Supplementary table 1** Baseline characteristics of renal death

| Characteristics | Overall | Quartile 1 | Quartile 2 | Quartile 3 | Quartile 4 | P value |
| --- | --- | --- | --- | --- | --- | --- |
|  | (N=3,975) | (N=993) | (N=994) | (N=994) | (N=994) |  |
| Renal death | 70 (1.76) | 12 (1.21) | 12 (1.21) | 21 (2.11) | 25 (2.52) | 0.06 |

**Supplementary table 2** Cox proportion hazard model stratified by 4 groups according to the IBI scores into quartiles for renal mortality.

| **Models** | **Model 1** | | **Model 2** | | **Model 3** | | **Model 4** | | **Model 5** | | **Model 6** | |
| --- | --- | --- | --- | --- | --- | --- | --- | --- | --- | --- | --- | --- |
|  | **HR (95%CI)** | **P-Value** | **HR (95%CI)** | **P-Value** | **HR (95%CI)** | **P-Value** | **HR (95%CI)** | **P-Value** | **HR (95%CI)** | **P-Value** | **HR (95%CI)** | **P-Value** |
| **Renal mortality** | | | | | | | | |  |  |  |  |
| **IBI was analyzed as a continuous variable** | | | | | | | | |  |  |  |  |
| Per 1-unit increment | 1.24 (1.06-1.44) | 0.006 | 1.29 (1.11-1.5) | 0.001 | 1.32 (1.13-1.55) | 0.001 | 1.27 (1.08-1.5) | 0.004 | 1.27 (1.06-1.52) | 0.009 | 1.18 (0.99-1.42) | 0.07 |
| **IBI was analyzed as a** **categorical variable** | | | | | | | | |  |  |  |  |
| Quartile 1 | Ref | - | Ref | - | Ref | - | Ref | - | Ref | - | Ref | - |
| Quartile 2 | 1.04 (0.47-2.31) | 0.93 | 1.06 (0.48-2.36) | 0.89 | 0.98 (0.41-2.31) | 0.96 | 1.00 (0.42-2.35) | 0.99 | 1.11 (0.46-2.68) | 0.82 | 0.93 (0.39-2.21) | 0.87 |
| Quartile 3 | 1.85 (0.91-3.76) | 0.09 | 1.85 (0.91-3.76) | 0.09 | 1.83 (0.86-3.88) | 0.12 | 1.89 (0.89-4.01) | 0.10 | 1.82 (0.82-4.04) | 0.14 | 1.55 (0.72-3.34) | 0.26 |
| Quartile 4 | 2.29 (1.15-4.56) | 0.02 | 2.71 (1.36-5.4) | 0.005 | 2.90 (1.39-6.02) | 0.004 | 2.47 (1.17-5.19) | 0.02 | 2.41 (1.10-5.28) | 0.03 | 1.68 (0.77-3.67) | 0.19 |
| P for trend | 0.005 | | 0.001 | | 0.001 | | 0.005 | | 0.01 | | 0.10 | |

**Abbreviation:** IBI, inflammatory burden index.

Model 1: unadjusted.

Model 2: adjusted for age and gender.

Model 3: further adjusted for BMI, race and education.

Model 4: further adjusted for congestive heart failure, coronary artery disease, hypertension, diabetes mellitus and stroke.

Model 5: further adjusted for uACR classification based on model 4.

Model 6: further adjusted for eGFR classification based on model 4.

**Supplementary table 3** Cox proportion hazard model stratified by 4 groups according to the IBI scores into quartiles for all-cause mortality after excluding participants with dialysis.

| **Models** | **Model 1** | | **Model 2** | | **Model 3** | | **Model 4** | | **Model 5** | | **Model 6** | |
| --- | --- | --- | --- | --- | --- | --- | --- | --- | --- | --- | --- | --- |
|  | **HR (95%CI)** | **P-Value** | **HR (95%CI)** | **P-Value** | **HR (95%CI)** | **P-Value** | **HR (95%CI)** | **P-Value** | **HR (95%CI)** | **P-Value** | **HR (95%CI)** | **P-Value** |
| **All-cause mortality** | | | | | | | | |  |  |  |  |
| **IBI was analyzed as a continuous variable** | | | | | | | | |  |  |  |  |
| Per 1-unit increment | 1.24 (1.06-1.44) | 0.006 | 1.29 (1.11-1.5) | 0.001 | 1.32 (1.13-1.55) | 0.001 | 1.27 (1.08-1.5) | 0.004 | 1.12 (1.08-1.17) | <0.001 | 1.12 (1.08-1.16) | <0.001 |
| **IBI was analyzed as a categorical variable** | | | | | | | | |  |  |  |  |
| Quartile 1 | Ref | - | Ref | - | Ref | - | Ref | - | Ref | - | Ref | - |
| Quartile 2 | 1.17 (1.03-1.33) | 0.017 | 1.22 (1.07-1.39) | 0.003 | 1.22 (1.07-1.4) | 0.003 | 1.22 (1.07-1.4) | 0.004 | 1.22 (1.06-1.4) | 0.004 | 1.19 (1.04-1.36) | 0.01 |
| Quartile 3 | 1.25 (1.1-1.42) | 0.001 | 1.25 (1.1-1.42) | 0.001 | 1.26 (1.1-1.44) | 0.001 | 1.28 (1.12-1.46) | <0.001 | 1.27 (1.11-1.46) | <0.001 | 1.27 (1.11-1.45) | 0.001 |
| Quartile 4 | 1.37 (1.21-1.55) | <0.001 | 1.67 (1.47-1.89) | <0.001 | 1.64 (1.43-1.87) | <0.001 | 1.6 (1.4-1.84) | <0.001 | 1.59 (1.38-1.82) | <0.001 | 1.55 (1.35-1.78) | <0.001 |
| P for trend | <0.001 | | <0.001 | | <0.001 | | <0.001 | | <0.001 | | <0.001 | |

**Abbreviation:** IBI, inflammatory burden index.

Model 1: unadjusted.

Model 2: adjusted for age and gender.

Model 3: further adjusted for BMI, race and education.

Model 4: further adjusted for congestive heart failure, coronary artery disease, hypertension, diabetes mellitus and stroke.

Model 5: further adjusted for uACR classification based on model 4.

Model 6: further adjusted for eGFR classification based on model 4.

**Supplementary table 4** Cox proportion hazard model stratified by 4 groups according to the IBI scores into quartiles for cardiovascular mortality after excluding participants with dialysis.

| **Models** | **Model 1** | | **Model 2** | | **Model 3** | | **Model 4** | | **Model 5** | | **Model 6** | |
| --- | --- | --- | --- | --- | --- | --- | --- | --- | --- | --- | --- | --- |
|  | **HR (95%CI)** | **P-Value** | **HR (95%CI)** | **P-Value** | **HR (95%CI)** | **P-Value** | **HR (95%CI)** | **P-Value** | **HR (95%CI)** | **P-Value** | **HR (95%CI)** | **P-Value** |
| **Cardiovascular mortality** | | | | | | | | |  |  |  |  |
| **IBI was analyzed as a continuous variable** | | | | | | | | |  |  |  |  |
| Per 1-unit increment | 1.09 (1.02-1.15) | 0.007 | 1.16 (1.09-1.23) | <0.001 | 1.15 (1.08-1.22) | <0.001 | 1.13 (1.06-1.20) | <0.001 | 1.11 (1.04-1.18) | 0.002 | 1.11 (1.05-1.19) | 0.001 |
| **IBI was analyzed as a categorical variable** | | | | | | | | |  |  |  |  |
| Quartile 1 | Ref | - | Ref | - | Ref | - | Ref | - | Ref | - | Ref | - |
| Quartile 2 | 1.21 (0.96-1.53) | 0.10 | 1.27 (1.01-1.6) | 0.04 | 1.25 (0.99-1.59) | 0.07 | 1.26 (0.99-1.61) | 0.06 | 1.23 (0.97-1.58) | 0.09 | 1.22 (0.96-1.56) | 0.11 |
| Quartile 3 | 1.28 (1.01-1.61) | 0.04 | 1.28 (1.02-1.61) | 0.04 | 1.27 (1.01-1.62) | 0.04 | 1.26 (0.99-1.61) | 0.06 | 1.23 (0.97-1.58) | 0.09 | 1.24 (0.97-1.58) | 0.09 |
| Quartile 4 | 1.45 (1.16-1.81) | 0.001 | 1.78 (1.42-2.23) | <0.001 | 1.71 (1.34-2.17) | <0.001 | 1.65 (1.29-2.10) | <0.001 | 1.58 (1.23-2.02) | <0.001 | 1.59 (1.25-2.04) | <0.001 |
| P for trend | 0.001 | | <0.001 | | <0.001 | | <0.001 | | <0.001 | | <0.001 | |

**Abbreviation:** IBI, inflammatory burden index.

Model 1: unadjusted.

Model 2: adjusted for age and gender.

Model 3: further adjusted for BMI, race and education.

Model 4: further adjusted for congestive heart failure, coronary artery disease, hypertension, diabetes mellitus and stroke.

Model 5: further adjusted for uACR classification based on model 4.

Model 6: further adjusted for eGFR classification based on model 4.

**Supplementary table 5** Cox proportion hazard model stratified by 4 groups according to the IBI scores into quartiles for renal mortality after excluding participants with dialysis.

| **Models** | **Model 1** | | **Model 2** | | **Model 3** | | **Model 4** | | **Model 5** | | **Model 6** | |
| --- | --- | --- | --- | --- | --- | --- | --- | --- | --- | --- | --- | --- |
|  | **HR (95%CI)** | **P-Value** | **HR (95%CI)** | **P-Value** | **HR (95%CI)** | **P-Value** | **HR (95%CI)** | **P-Value** | **HR (95%CI)** | **P-Value** | **HR (95%CI)** | **P-Value** |
| **Renal mortality** | | | | | | | | |  |  |  |  |
| **IBI was analyzed as a continuous variable** | | | | | | | | |  |  |  |  |
| Per 1-unit increment | 1.17 (0.99-1.4) | 0.07 | 1.24 (1.04-1.47) | 0.01 | 1.26 (1.06-1.51) | 0.01 | 1.22 (1.01-1.47) | 0.04 | 1.23 (1.01-1.5) | 0.04 | 1.16 (0.96-1.42) | 0.13 |
| **IBI was analyzed as a categorical variable** | | | | | | | | |  |  |  |  |
| Quartile 1 | Ref | - | Ref | - | Ref | - | Ref | - | Ref | - | Ref | - |
| Quartile 2 | 1.13 (0.5-2.56) | 0.77 | 1.16 (0.51-2.63) | 0.72 | 1.06 (0.44-2.57) | 0.89 | 1.11 (0.46-2.69) | 0.81 | 1.10 (0.46-2.66) | 0.83 | 1.04 (0.43-2.51) | 0.94 |
| Quartile 3 | 1.83 (0.87-3.84) | 0.11 | 1.82 (0.87-3.83) | 0.11 | 1.79 (0.81-3.95) | 0.15 | 1.9 (0.86-4.2) | 0.11 | 1.69 (0.75-3.77) | 0.20 | 1.56 (0.7-3.5) | 0.28 |
| Quartile 4 | 2.02 (0.97-4.23) | 0.06 | 2.44 (1.17-5.11) | 0.02 | 2.63 (1.2-5.77) | 0.02 | 2.28 (1.02-5.1) | 0.04 | 2.14 (0.95-4.81) | 0.07 | 1.77 (0.77-4.06) | 0.18 |
| P for trend | 0.03 | | 0.007 | | 0.006 | | 0.02 | | 0.04 | | 0.11 | |

**Abbreviation:** IBI, inflammatory burden index.

Model 1: unadjusted.

Model 2: adjusted for age and gender.

Model 3: further adjusted for BMI, race and education.

Model 4: further adjusted for congestive heart failure, coronary artery disease, hypertension, diabetes mellitus and stroke.

Model 5: further adjusted for uACR classification based on model 4.

Model 6: further adjusted for eGFR classification based on model 4.

**Supplementary table 6** Comparison of C-statistics for concordance index (C-index) between IBI-based and CRP-only-based multivariate models.

|  | IBI was analyzed as a categorical variable | IBI was analyzed as a continuous variable | CRP | difference of C-index* | difference of C-index** |
| --- | --- | --- | --- | --- | --- |
|  | C-index (95% CI) | C-index (95% CI) | C-index (95% CI) | P-value | P-value |
| All-cause mortality | 0.773 (0.763-0.784) | 0.773 (0.762-0.783) | 0.681 (0.668-0.693) | <0.001 | <0.001 |
| Cardiovascular mortality | 0.797 (0.779-0.814) | 0.795 (0.778-0.813) | 0.712 (0.690-0.733) | <0.001 | <0.001 |

Abbreviation: IBI, inflammatory burden index; CRP, C-reactive protein; C-index, Concordance index.

* IBI-based model vs CRP-based model. Covariates include age, gender, BMI, race, education, congestive heart failure, coronary artery disease, hypertension, diabetes mellitus and stroke. IBI was analyzed as a graded variable.

** IBI-based model vs CRP-based model. Covariates include age, gender, BMI, race, education, congestive heart failure, coronary artery disease, hypertension, diabetes mellitus and stroke. IBI was analyzed as a continuous variable.
